# The absence of cruelty is not the presence of humanness: physicians and the death penalty in the United States

**DOI:** 10.1186/1747-5341-7-13

**Published:** 2012-12-03

**Authors:** Joel B Zivot

**Affiliations:** 1 Department of Anesthesiology, Emory University, 201 Dowman Drive, Atlanta, GA, 30322, USA

## Abstract

The death penalty by lethal injection is a legal punishment in the United States. Sodium Thiopental, once used in the death penalty cocktail, is no longer available for use in the United States as a consequence of this association. Anesthesiologists possess knowledge of Sodium Thiopental and possible chemical alternatives. Further, lethal injection has the look and feel of a medical act thereby encouraging physician participation and comment. Concern has been raised that the death penalty by lethal injection, is cruel. Physicians are ethically directed to prevent cruelty within the doctor-patient relationship and ethically prohibited from participation in any component of the death penalty. The US Supreme Court ruled that the death penalty is not cruel per se and is not in conflict with the 8th amendment of the US constitution. If the death penalty is not cruel, it requires no further refinement. If, on the other hand, the death penalty is in fact cruel, physicians have no mandate outside of the doctor patient relationship to reduce cruelty. Any intervention in the name of cruelty reduction, in the setting of lethal injection, does not lead to a more humane form of punishment. If physicians contend that the death penalty can be botched, they wrongly direct that it can be improved. The death penalty cocktail, as a method to reduce suffering during execution, is an unverifiable claim. At best, anesthetics produce an outward appearance of calmness only and do not address suffering as a consequence of the anticipation of death on the part of the condemned.

## 

Sodium thiopental, a drug once standard in the practice of anesthesiology, is no longer available in the USA. This is due to concerns by the manufacturer over use in the death penalty via lethal injection. ^a^Anesthesiologists possess the pharmacological and technical expertise required to utilize alternatives to sodium thiopental injection in the setting of medical practice. From a technical and pharmacological perspective, the death penalty, by lethal injection, appears to possess common elements to the practice of Anesthesiology. As a consequence, death penalty proponents have sought advice from anesthesiologists and derive benefit both from the applicable knowledge possessed in the medical practitioner and the ability to usurp a civilized image by association. Death penalty opponents have used the 8th amendment of the US constitution as justification against the death penalty [1,2]. The argument asserts that death by injection would constitute cruel and unusual punishment. Indeed, evidence exists that the death penalty by lethal injection, as practiced in the United States, falls below the standard of veterinary euthanasia [3] or the normal conduct of an anesthetic performed within a medical setting [4]. States that practice the death penalty have attempted to answer this concern by asserting that the death penalty is in fact constitutional by imposing a standard of humanness [5]. This paper will address the following concerns: First, what is meant by cruelty in the context of the death penalty? Second, what are the moral duties and obligations of the physician, both as doctor and citizen, with respect to conduct in society? Last, what is the role of the physician with respect to mitigation of cruelty and promotion of humanness in the setting of the death penalty?

It is important to draw the distinction between cruel acts and cruel individuals. When we say that a person is “cruel” we are referring to their motives. They want to inflict pain on others, take pleasure in the pain of others, or are indifferent to the pain of others. When we say that an action is cruel we are referring to its consequences: it causes unnecessary or excessive pain. Cruel people are prone to engage in cruel practices but sometimes kind and gentle people engage in cruel practices from a professed motive of mercy: they want to diminish the pain/suffering involved in the cruel practices. Cruel punishment was defined by the original framers of the constitution according to the prevailing notions of the time. The legal system recognizes that cruelty will always reflect a standard commensurate with the maturation of a civil society and that punishment should be proportionate to the severity of the crime. The U.S. Supreme Court has held that the death penalty itself is not inherently cruel, but has described it as “an extreme sanction, suitable to the most extreme of crimes” [6] It is important to recognize that constitutional protection is concerned with the method of punishment, not what is considered as the necessary suffering inherent in any method utilized to end a life humanely [7]. The court has considered the death penalty from a consequentialist perspective, that is, fundamentally, the death penalty is successful when the result is death of the condemned. From time to time, as society evolves, the court will evaluate the method of execution against the current cruelty standard only, not the rightness or wrongness of the death penalty.

In the setting of the doctor-patient relationship, medical ethics directs the physician to act without maleficence, that is, to do no harm. Is it reasonable that a physician, acting in ones own professional capacity, has no moral duty/obligation to anyone other than the patient? Many would argue that physicians have multiple other obligations, e.g., to public health and safety, to obey the law, the duty to warn, the duty to report, and various other public-spirited duties. On occasion, military physicians have duties that potentially place them in situations where medical ethics and military interests collide. Physicians’ desire to reduce cruelty in the setting of the death penalty may be compared to the actions of military physicians’ who use medical knowledge to enhance prisoner interrogation, resolve hunger strikes and prescribe psychotropic medications to retain soldiers in combat areas or accelerate a return to active duty [8]. Rather than affirming the universal ethical duties of physicians, recent Department of Defense memoranda create vagueness by distinguishing treating from non-treating physicians, [9] in order to justify participation of non-treating physicians in using their medical knowledge to inflict cruelty. The American Medical Association, Council on Ethical and Judicial Affairs, adopted the World Medical Association Declaration of Tokyo (1975) [10] which refutes the claim that physician participation in torture or other coercive, non-therapeutic activities benefits the detainee by affording some form of protection [11]. Physicians are citizens, but in a free society, the adherence to a rule is not inviolate. The conduct of a citizen allows thoughtful dissent from certain activities. A physician may refuse to perform certain military duties as a form of conscientious objection. With regard to the death penalty, physician refusal carries a higher moral authority than participatory complicity. Moral self-deception is created when a small purpose close at hand interferes with a greater purpose, perhaps more distant [12].

David Waisel makes the case for physician involvement in the death penalty by lethal injection from the perspective of “humanness [13].” He refers to numerous reports of executions that proceeded with difficulties including problems with intravenous access, [10,11] subjective assessments by observers that suffering occurred in the condemned, [14] and drug and dosage errors [15]. The claim that the death penalty by lethal injection can be botched suggests that it can therefore be improved. The appeal for improvement in the name of humaneness succeeds in drawing physicians in, [16] by appealing to a sympathetic concern for the welfare of others. From the above considerations of ethics and cruelty, the argument in support of humanness fails for several reasons. Physicians who participate in the death penalty are not concerned with prolonging life. This would certainly be the basic activity of medical practice. Physician participation then is in the name of mercy, or a reduction in the cruelty of lethal injection, except when it addresses that the purpose of the injection is to produce death. How cruel are the details of lethal injection apart from the lethality itself? By how much does a doctor’s intervention reduce cruelty during execution? Non-physicians can establish intravenous access, and are able to draw up and inject medication. Non-physicians can provide comfort to the condemned as they anticipate and finally approach the execution table. It is conceivable that physician participation might increase cruelty from the perspective of the condemned. Physician endorsement of execution is so counter to normal medical practice that in the prisoners final moments, all vestiges of hope of a better society, should that be imagined, would be lost. Ultimately, the assertion that physician participation reduces cruelty is unverifiable. Only outwardly does it seem so by the witnesses. The administration of the death penalty is absolutely silent on the experience of the witness and needs not be addressed further.

Physicians are ethically directed to act with beneficence, and humanness may be subsumed within beneficence. Beneficence and humanness, as acts of conduct by physicians, are only directives within the doctor-patient relationship. Though acting humanely as a general activity may benefit society, it is not enforceable as a general standard of human conduct. If it is asserted that physicians are required to perform humane actions outside of the doctor-patient relationship, operationalizing such activity would be impossible. Within the complete rendering of human affairs, much inhumanity exists. No method exists to rank order humane tasks yet some method of humane triage would be required. If physicians position themselves as possessing statutory requirement for humane intervention in all affairs, they would otherwise be rightly accused of acting in one area at the seemingly arbitrary, or value laden, neglect of another. Physicians, like all citizens, may choose to act with humanity. Physicians may claim that in certain circumstances, they are not acting as a physician but as a private citizen.

Arguing that within the context of the death penalty the physician is a private citizen acting with “humanness” is flawed. Physician involvement is sanctioned by the state because physicians posses the medical knowledge of the components of lethal injection. Physicians, however, are not able to separate their medical knowledge and conduct in circumstances that possess the look and feel of a medical act. The death penalty does not claim to be a medical act and is therefore not subject to the standards within the performance of medical acts. Yet, it has chosen to usurp the tools of the medical trade thereby misleading physicians to believe they are working within the framework of medicine, and the public to believe that civility and safe oversight are in place.

Physicians are unambiguously prohibited from active participation in the death penalty according to the American Medical Associations opinion on capital punishment [17]. In the United States, only 20% of physicians are members of the AMA [18]. Additionally, only 7 of the 35 states that use the death penalty have statutory or regulatory incorporation of AMA ethical guidelines [19]. States have successfully barred medical boards from disciplining physicians who have been involved with the death penalty [20]. The AMA is limited in ability to punish physicians who are at odds with AMA policy beyond revocation of AMA membership. AMA membership is not a requirement by physicians to obtain medical licensure or practice medicine. State governments affirm legal authority in the regulation of medical practice, even in circumstance where the state medical board objects. In this regard, medical ethical conduct and state legal authority are at odds. The Nuremberg defense has clearly defined that medical practice, outside of ethical conduct is not made right by state fiat [21].

The death penalty by lethal injection is a two-fold process. First, a state government acquires a chemical, or a combination of chemicals that when injected, causes death in people. Second, these chemicals are given as a punishment to individuals who have been lawfully convicted of certain offences with the purpose of causing them to die. In this situation, the convicted individual is not a patient and therefore physicians have no role in this activity. Physicians are neither capable nor required to remove cruelty in circumstances outside of the doctor-patient relationship. Physicians as citizens are not charged with the promotion of humanness outside of the practice of medicine. Physicians therefore have no obligation or mandate to be involved. It remains the states prerogative to execute individuals but it should be prohibited from using words or methods that are terms of art, which are used by physicians to describe medical practice.

In summary, physicians have no ethical requirement to participate in the death penalty. Fundamentally, any invocation of a reduction in suffering consequent to physician activity should exist within a doctor-patient relationship. A physician and a condemned prisoner have no doctor-patient relationship in the context of the administration of the death penalty by lethal injection. If, according to the United States Supreme Court, the death penalty is not cruel per se, it needs no improvement. If the death penalty is cruel, then attempts to reduce cruelty by pharmacological adjustments are not necessarily humane, or worse, create an illusion of humanness as they are physician directed.

## Endnotes

^a^http://www.ashp.org/drugshortages/current/bulletin.aspx?id=563.
